# AGEISM, CONTACT, AND OLDER ADULT ADLS RELATE TO MENTAL HEALTH TRAINEES’ INTEREST IN GEROPSYCHOLOGY

**DOI:** 10.1093/geroni/igac059.1840

**Published:** 2022-12-20

**Authors:** Grace I L Caskie, Abigail Voelkner, MaryAnn Sutton, Anastasia Canell, Eve Root

**Affiliations:** Lehigh University, Bethlehem, Pennsylvania, United States; Lehigh University, Bethlehem, Pennsylvania, United States; UPMC Psychological Associates, Harrisburg, Pennsylvania, United States; Lehigh University, Mineola, New York, United States; Lehigh University, Bethlehem, Pennsylvania, United States

## Abstract

Despite growing demand, few mental healthcare professionals specialize in clinical work with older adults. A better understanding of factors related to geropsychology interest may increase the pipeline of future geropsychologists. Graduate-level trainees (N=460; 67.4% doctoral; age=21-64) completed the Fraboni Scale of Ageism, Contact with Older Adults Scale, six indicators of interest in work with older adults, and an imagined “typical” older adult’s ability to complete activities of daily living (ADLs). 60% imagined full ADL independence. Between 14.1%-25.7% expressed strong interest in education/training related to treating older adults and having some older adult clients; only 6.7% planned to specialize in clinical work with older adults. In regression analyses (R-squared=16%-32%), more ageist attitudes, less contact, and being a master’s trainee were related to less interest. ADL status was significant only for interest in specialization; imagining more ADL-dependence related to higher interest in specializing in older adults. ADL status significantly moderated the relation of contact to interest in learning about issues related to older adults; ADL-based differences in interest were non-significant at low and average contact, but at high contact, interest in learning about older adults was significantly higher when the older adult was imagined as ADL-dependent rather than ADL-independent. Findings may indicate benevolent ageism partially motivates trainees’ interest in learning about/working with older adults whom they imagine need more help with basic tasks of daily living. Increasing contact, reducing ageist attitudes, and providing more clinical opportunities with older adults may facilitate trainees’ readiness and interest in future clinical work with older adults.

